# Dexmedetomidine ameliorates gut lactate production and impairment of exogenous lactate clearance in an endotoxic sheep model

**DOI:** 10.1186/2197-425X-3-S1-A414

**Published:** 2015-10-01

**Authors:** G Hernandez, P Tapia, G Ospina-Tascón, A Bruhn, D Soto, L Alegría, N Jarufe, C Luengo, R Menchaca, A Meissner, MI Vives, J Bakker

**Affiliations:** Pontificia Universidad Catolica de Chile, Santiago, Chile; Fundación Valle del Lili, Cali, Colombia; Universidad de Chile, Santiago, Chile; Erasmus MC University Medical Center, Rotterdam, the Netherlands

## Introduction

The mechanisms of persistent hyperlactemia during endotoxic shock are probably multifactorial. Both hypoperfusion-related anaerobic production and adrenergic-driven aerobic generation have been implicated. More recently an early and severe impairment in exogenous lactate clearance has also been described [1]. Theoretically, an excessive adrenergic response could influence all these mechanisms and thus aggravate the problem.

## Objectives

To assess the effects of dexmedetomidine (DEX) on lactate production and exogenous lactate clearance in an endotoxic shock model.

## Methods

**Figure 1:**
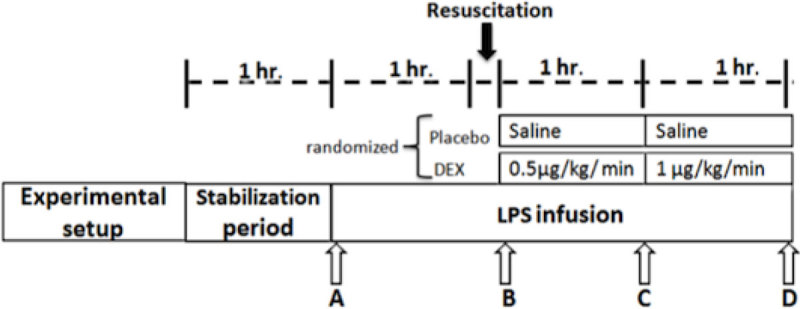
Figure 1

Twelve anesthetized sheep were subjected to a multimodal hemodynamic/perfusion assessment including hepatic and portal vein catheterizations, total hepatic blood flow, sublingual microcirculation, and muscle microdialysis. After the monitoring phase, all received a 5 mcg/kg LPS bolus (E coli O127:B8®) and then 4 mcg·kg^1^·hr^1^ for the rest of the experiment. After 1hr they were volume resuscitated, and then randomized to placebo or DEX. Sampling and exogenous lactate clearances [2] were performed at 4 points (figure 1).

## Results

DEX was not associated with any adverse hemodynamic effect in terms of cardiac output, heart rate, mixed venous oxygen saturation, central venous-arterial pCO_2_ gradient and NE requirements as compared to placebo. DEX animals presented significant lower epinephrine levels (4.6 ± 1.3 vs 7.1 ± 1.4 ng/ml), arterial lactate levels (6.4 ± 3.1 vs 9.2 ± 1.8 mmol/l), portal vein lactate levels (6.2 ± 2.2 vs 8.1 ± 2.0 mmol/l); and higher portal vein O2 saturations (78 ± 16 vs 68 ± 11 %), and exogenous lactate clearance (7.2 ± 5.4 vs 2.9 ± 1.5 ml/kg/min) as compared to placebo at point D. No differences in muscle lactate production or sublingual microcirculatory parameters could be observed.

## Conclusions

Dexmedetomidine ameliorates the increase in gut lactate production and impairment of exogenous lactate clearance in experimental endotoxic shock. This effect is associated with a significant reduction in systemic epinephrine levels.

## Grant Acknowledgment

FONDECYT 1130200, Chile
